# Supplementary material for: Stk10 Deficiency in Mice Promotes Tumor Growth by Dysregulating the Tumor Microenvironment
Source: Biology (Basel). 2022 Nov 15;11(11):1668. doi: 10.3390/biology11111668 (PMC9687870; doi:10.3390/biology11111668)
Supplement: Supplementary file 1 [file biology-11-01668-s001.zip › biology-1916013-supplementary.pdf]

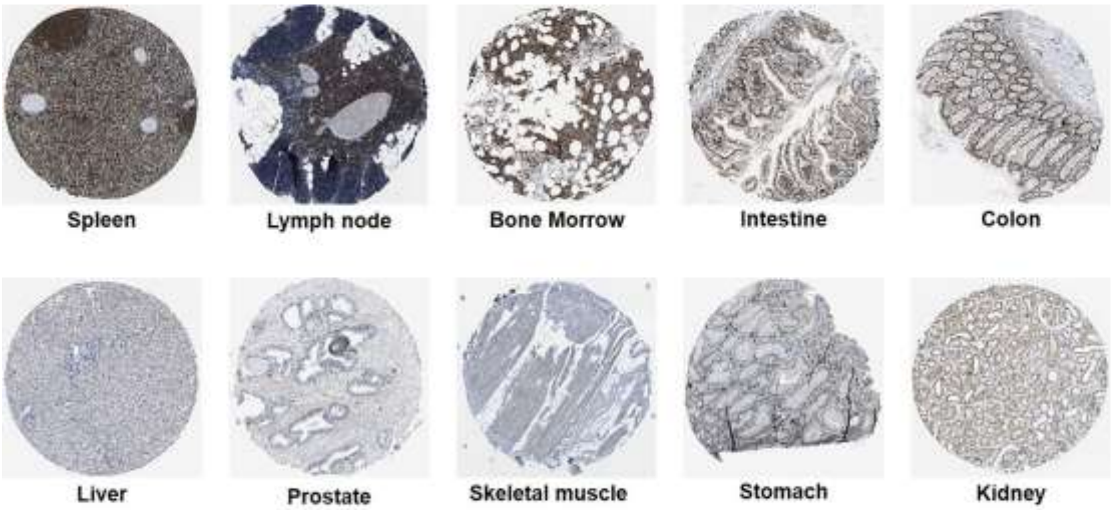


**Figure S1.** IHC data form The Human Protein Atlas showed that STK10 dominantly expressed in immune organs. In the IHC figure, STK10 (brown) was high expression in spleen, lymph node, bone morrow but low in liver, skeletal muscle, stomach.

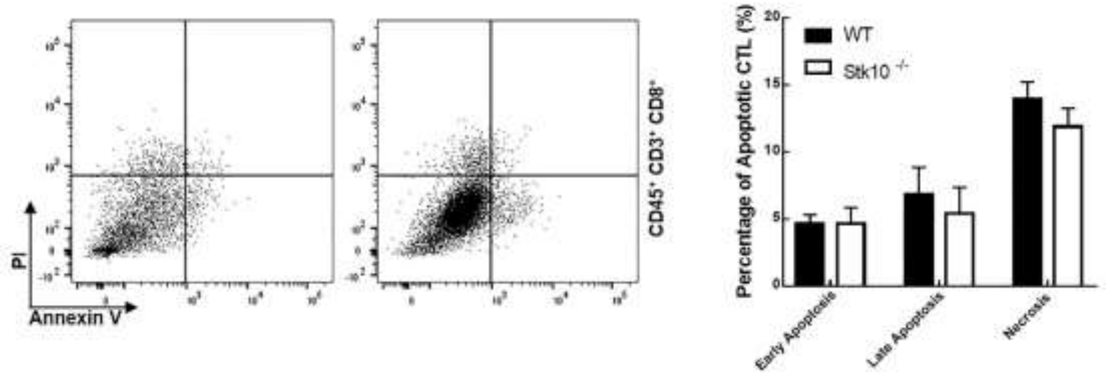


**Figure S2.** Effects of *Stk10* knockout on the apoptosis of CTLs. Early apoptosis: Annexin $V^{+}/PI^{-}$, Late apoptosis: Annexin $V^{+}/PI^{+}$, necrosis: Annexin $V^{-}/PI^{+}$. *p<0.05; **p<0.01.

**Table S1.** The primers were used in this study.

| Primers | Sequences (5'-3') |
|---|---|
| Stk10-test-F1 | TGTCGAGCCCAGAGTTGTATTGT |
| Stk10-test-R1 | ACGGCGGGGACGGAGAGTA |
| Stk10-*mus*-genetyping-F | TGTCGAGCCCAGAGTTGTATTGT |
| Stk10-*mus*-genetyping-R | ACGGCGGGGACGGAGAGTA |
| LOK-qRT-PCR-F | CGCATGGCCATGTACAAGAAGAGC |
| LOK-qRT-PCR-R | CTCGCATCTGGTTCTCGTGTTTCTG |
| GAPDH-F | CAGCCTCGTCCCGTAGACA |
| GAPDH-R | CGCTCCTGGAAGATGGTGAT |

F: forward primers, R: reverse primers.

**Table S2.** The antibodies were employed in this study.

| Antibodies | Source | Nation | Code number | Application |
|---|---|---|---|---|
| anti-STK10 | Abcam | UK | ab70484 | WB(1:1000) |
| anti-STK10 | Abcam | UK | Ab228802 | IHC (1:100) |
| anti-p-ERM | CST | USA | 3762s | WB (1:1000) |
| anti-Radixin | Abcam | UK | ab52495 | WB (1:1000) |
| anti-Ezrin | Abcam | UK | ab40839 | WB (1:1000) |
| anti-Moesin | Abcam | UK | ab52490 | WB(1:1000) |
| anti-GAPDH | BBI | China | 14793 | WB (1:1000) |

| | | | | |
|---|---|---|---|---|
| anti-Cleaved-caspase3 | CST | USA | 9661s | IHC (1:100) |
| anti-KI67 | ABclonal | China | A11390 | IHC (1:100) |
| IRDye800CW Donkey anti-Rabbit IgG | LI-COR | USA | 925-32213 | WB(1:10000) |
| DAPI | Sigma | USA | D9542 | IF/IHC(5ug/mL) |
| Goat anti-rabbit-594 | Invitrogen | USA | A-11012 | IF/IHC(1:500) |
| Goat anti-mouse-488 | Invitrogen | USA | A-11008 | IF/IHC(1:500) |
| anti-CD31 | Abcam | UK | ab182981 | IF (1:1000) |
| anti-CD45-FITC | eBioscience | USA | 14-0451-82 | 0.1ug/$10^6$ cells |
| anti-B220-V450 | eBioscience | USA | 12-0691-82 | 0.1ug/$10^6$ cells |
| anti-CD3-APC | Biolegend | USA | 100235 | 0.1ug/$10^6$ cells |
| anti-CD8-APC-eFluor780 | eBioscience | USA | 12-0621-82 | 0.1ug/$10^6$ cells |
| anti-CD69-V450 | eBioscience | USA | 12-0691-82 | 0.1ug/$10^6$ cells |
| anti-CD44-PE/Cyanine7 | eBioscience | USA | 12-0441-82 | 0.1ug/$10^6$ cells |
| anti-CD62L-APC | Biolegnd | USA | 104411 | 0.1ug/$10^6$ cells |
| anti-CTLA4- | Biolegnd | USA | 106313 | 0.1ug/$10^6$ cells |

| | | | | |
|---|---|---|---|---|
| PE/Cyanine7 | | | | |
| anti-PD1-V450 | eBioscience | USA | 11-9985-82 | 0.1ug/$10^6$ cells |
| anti-CD11b-APC | eBioscience | USA | 25-5941-82 | 0.1ug/$10^6$ cells |
| anti-NK1.1-PE | eBioscience | USA | 25-0452-82 | 0.1ug/$10^6$ cells |
| anti-Ly6G/6C (Gr-1)-PE | eBioscience | USA | 12-5931-81 | 0.1ug/$10^6$ cells |
| anti-F4/80-FITC | eBioscience | USA | 11-4801-85 | 0.1ug/$10^6$ cells |

CST, Cell signaling technology; BBI, Sangon Biotech; IF, Immunofluorescence; WB, Western Blotting; IHC, Immunohistochemistry.
